# Short Stimulus, Long Response: Sodium and Calcium Dynamics Explain Persistent Neuronal Firing

**DOI:** 10.1371/journal.pbio.1002320

**Published:** 2015-12-16

**Authors:** Richard Robinson

**Affiliations:** Freelance Science Writer, Sherborn, Massachusetts, United States of America

## Abstract

A new study shows that the prolonged firing of some neurons in response to a transient signal can be caused by a high intracellular level of sodium ions, which slows the decay of the calcium spike. Read the Research Article.

A neuron fires in response to a stimulus, and in most cases, becomes refractory to further firing while returning to its resting state. In some neurons, however, a brief stimulus induces prolonged firing, lasting many seconds or even minutes. What mechanisms underlie this persistent response? One hypothesis has been that the neuron is linked into circuits that feed back onto it, providing a long-lasting secondary source of excitation. But models of such systems suggest they are much more susceptible to perturbation than is found in the neurons that actually exhibit prolonged firing.

One such set of neurons are the mitral cells of the accessory olfactory bulb (AOB). In rodents and many other mammals (though not humans), the AOB processes pheromonal stimuli received by the vomeronasal organ, helping to regulate social interactions. In a new study in *PLOS Biology*, Asaph Zylbertal and colleagues show that the prolonged firing of the AOB’s mitral cells is independent of circuit-level control and is instead due to an unusually slow decay in the intracellular concentration of sodium ions.

The authors have previously shown that mitral cells respond to a brief but intense stimulus by firing for up to several minutes. Here, they combined electrophysiology, cellular imaging, and computational modeling to tease apart the mechanism underlying that persistent firing.

Recall that a resting neuron maintains a variety of ionic gradients across its plasma membrane. Importantly, the concentration of sodium is high outside the cell and low inside. At the dendrite, stimulation of the neuron opens sodium channels and sodium ions come flooding into the cell. The influx of sodium (combined with other ion changes) depolarizes the membrane, a change that then propagates down the axon as an action potential. The dendritic sodium channels then close and sodium is pumped out of the cell, restoring the gradient and the resting membrane potential.

Among the other ions in flux during neuronal firing is calcium. By injecting a calcium-sensitive dye into the mitral cells, the authors showed that the level of calcium in the tip of a dendritic branch (the “tuft”) rose quickly in response to stimulation, and then decayed very slowly, a phenomenon not observed in other regions of the cell. Furthermore, they found that the level of calcium was dependent on the strength of the stimulus received by the neuron.

Intracellular calcium is removed by two mechanisms: a calcium pump, and a sodium-calcium exchanger, in which the intake of extracellular sodium is coupled with the extrusion of calcium. But the activity of the exchanger and, hence, the rate of calcium extrusion depends on the strength of the sodium gradient. That, in turn, depends on the density of the major sodium exporter, the sodium/potassium ATPase. Thus, the authors reasoned, at low enough densities of the ATPase, intracellular sodium could remain high enough to reduce the activity of the exchanger or even reverse it, like a revolving door spinning backwards. This reversal could account for the prolonged elevation of intracellular calcium, which in turn could explain the prolonged firing of the mitral cells.

To test this idea, they built a model involving three membrane proteins: the sodium/calcium exchanger, the calcium pump, and the sodium/potassium ATPase. By incorporating details of intracellular diffusion and the varying density of membrane proteins among cellular compartments, they showed that the intracellular sodium concentration was a major determinant of the slow decay in calcium levels. While the calcium pump removed calcium from the cell, the exchanger, driven by high sodium to run in reverse, let it back in (Fig 1). Critically, the effect depended on having relatively few sodium/potassium pumps available, in order to keep sodium elevated. The model predicted, and in vitro measurements confirmed, that blocking the exchanger allowed calcium to fall faster and abolished the prolonged firing of mitral cells.

**Fig 1:**
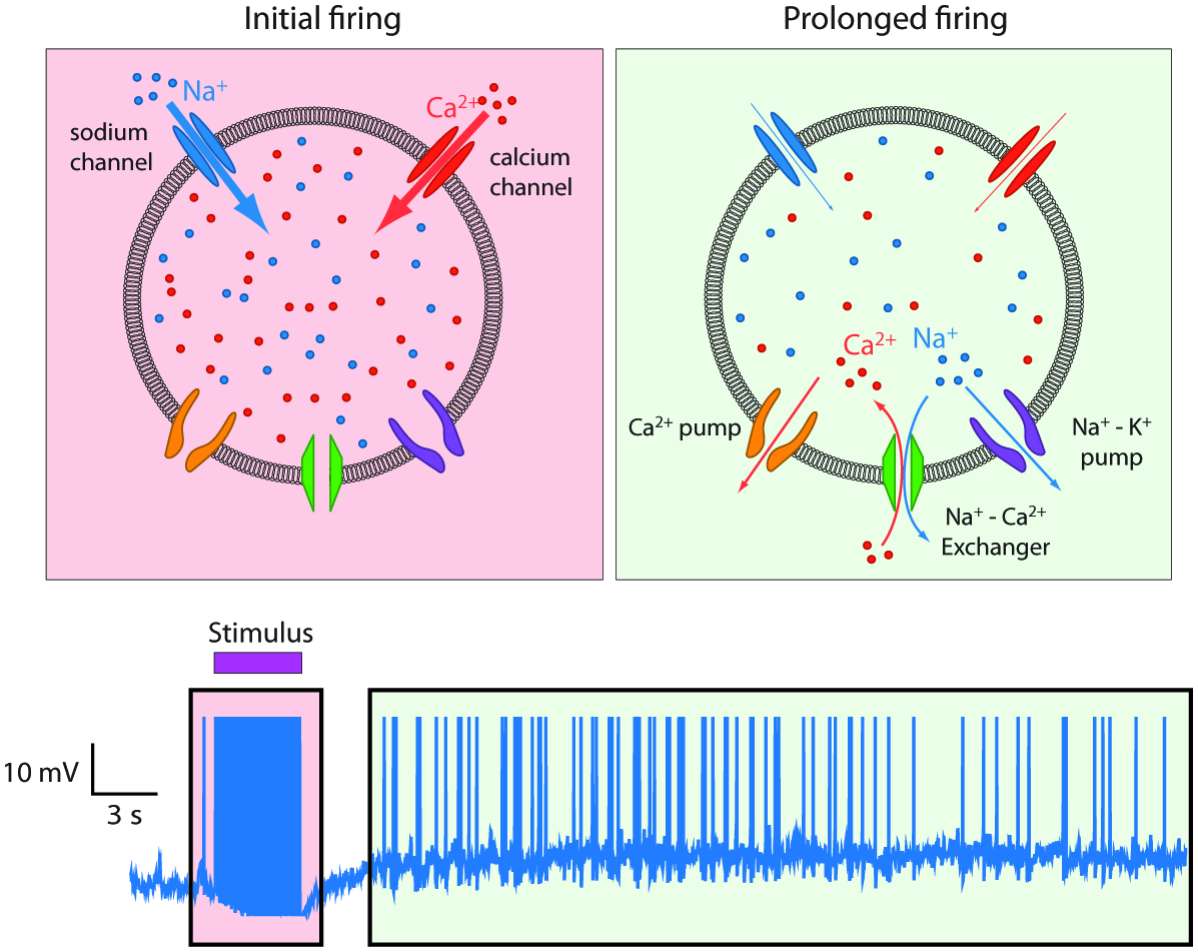
The ionic machinery behind prolonged firing. A schematic representation of a section in the dendritic tuft (top) showing the channels and transport mechanisms responsible for prolonged firing in AOB mitral cells (bottom). *Image credit*: *Asaph Zylbertal*.

The mechanism described here is unlikely to be limited to the accessory olfactory bulb’s mitral cells. Further experiments will test whether it also contributes to long-lasting firing in other brain areas, including those involved in working memory, which involves persistent firing in response to limited stimuli. In addition, given calcium’s critical role as an intracellular signaling molecule, activity-dependent prolonged elevation of calcium may contribute to changes in synaptic plasticity, gene expression, and other processes, at time scales previously unrecognized.
